# Screening High-Biomass Grasses for Cadmium Phytoremediation

**DOI:** 10.3390/plants13233450

**Published:** 2024-12-09

**Authors:** Olívia Bibiana Souza Dias, Lucélia Borgo, Deivisson Ferreira da Silva, Alisson de Carli Souza, Tiago Tezotto, Jaco Vangronsveld, Luiz Roberto Guimarães Guilherme, Flávio Henrique Silveira Rabêlo

**Affiliations:** 1 Department of Soil Science, Federal University of Lavras, Lavras 37200-900, Brazil; olivia.dias2@estudante.ufla.br (O.B.S.D.); borglucelia@gmail.com (L.B.); deivisson.silva@estudante.ufla.br (D.F.d.S.); alisson.souza2@estudante.ufla.br (A.d.C.S.); guilherm@ufla.br (L.R.G.G.); 2 Centre for Environmental Sciences, Hasselt University, 3590 Diepenbeek, Belgium; jaco.vangronsveld@uhasselt.be; 3 Federal Institute Catarinense, Araquari 89245-000, Brazil; 4 Luiz de Queiroz College of Agriculture, University of São Paulo, Piracicaba 13418-900, Brazil; tiago.tezotto@usp.br; 5 Department of Plant Physiology and Biophysics, Maria Curie Sklodowska University, 20-033 Lublin, Poland

**Keywords:** bioconcentration factor, biomass production, cadmium, nutritional disorders, phytoextraction, phytostabilization, poaceae, translocation factor

## Abstract

Investigating the ability of non-hyperaccumulator plants to grow in soils polluted by cadmium (Cd) and their potential for phytostabilization or phytoextraction is essential for assessing their use in phytomanagement efficiency. Therefore, we evaluated the tolerance of high-biomass grasses to Cd by measuring biomass production and element accumulation and valued them for their suitability for phytoextraction or phytostabilization purposes on moderately Cd-polluted land (total Cd concentration of 7.5 mg kg^−1^) by determining Cd accumulation in the plants and calculating the bioconcentration (Cd BCF) and translocation factors (Cd TF). Among the ten species under investigation, *Panicum maximum* cv. Massai and *Pennisetum glaucum* cv. Purpureum Schum showed lower root biomass due to Cd exposure. Cadmium exposure altered element accumulation in some grass species by reducing P, K, and Mg accumulation in *P. glaucum* cv. Purpureum Schum; K accumulation in *P. maximum* cv. Massai; Mg accumulation in *P. maximum* cv. Mombaça; Ca, Fe, and Zn accumulation in *P. maximum* cv. Aruana; and B accumulation in *Brachiaria brizantha* cv. Piatã. However, this was not correlated with lowered biomass production, except for K, which was associated with lowered root biomass allocation in *P. maximum* cv. Massai and *P. glaucum* cv. Purpureum Schum. Cadmium concentrations decreased from roots to shoots, indicating a clear limitation of upward Cd transport. Although some grasses exhibited a Cd BCF > 1, the Cd TF remained below 0.4 for all tested species. These results indicate that, under moderate Cd pollution, the evaluated grasses are more suitable for Cd phytostabilization than phytoextraction, except for *P. maximum* cv. Massai and *P. glaucum* cv. Purpureum Schum, which showed inhibited root growth and may not be efficient over time.

## 1. Introduction

Cadmium (Cd) is generally present in the environment at low levels; however, human activities have increased its levels in environmental media relevant to human exposure [1]. Environmental pollution by Cd is a serious socio-environmental problem that runs counter to the fulfillment of many of the United Nations’ Sustainable Development Goals (https://sdgs.un.org/goals (accessed on 8 December 2024)). This metal is classified as a human carcinogen and exerts toxic effects on the kidneys, as well as the skeletal and respiratory systems [1]. Therefore, it is essential to lower Cd concentrations in polluted environments. From an environmental and economic point of view, phytoremediation is a friendly technology that can contribute to reaching this objective [2,3]. However, this technology’s unsafe disposal of polluted biomass remains problematic, as it may cause secondary pollution. In this context, it is essential to enhance the effective recovery of Cd in biomass [1] through alternative economic valorization, such as bioenergy production [4,5,6]. It is necessary to highlight that plants with high biomass production are generally considered more viable candidates for bioenergy production [5].

Although hyperaccumulator plants are much more efficient at removing Cd from polluted environments than non-hyperaccumulators [7], the number of known Cd hyperaccumulators is low, and such species are generally adapted to specific climatic conditions that restrict their cultivation worldwide [8]. The use of Cd hyperaccumulators for phytoremediation in tropical regions is not feasible. Among the grass species evaluated for Cd phytoremediation in the tropics, grasses have shown promising results in environments with low to moderate Cd concentrations [7,9,10,11,12,13,14,15,16]. However, the time required by grasses to lower even low Cd concentrations to background reference values exceeds 20 years [17], which jeopardizes the applicability of this technology compared to others [18]. On the other hand, using high-biomass grasses could result in greater Cd exportation from the soil and increase the competitiveness of phytoremediation compared to other technologies, mainly by allowing bioenergy production and generation of high volumes of high-value by-products [5]. Between such grasses cultivated in tropical regions, the genera *Andropogon*, *Brachiaria* (Syn. *Urochloa*), *Panicum* (Syn. *Megathyrsus*), *Pennisetum* and *Sorghum* stand out due to ease of cultivation and rapid growth [19]. However, most grasses, if not all, possess mechanisms that restrict trace element (TE) uptake and translocation from roots to shoot [20], which often leads to higher TE accumulation in the underground parts of the plants, which can lead to imbalances of essential minerals [21]. Therefore, it is crucial to screen grasses for their potential use.

Theoretically, most grasses are more suitable for Cd phytostabilization due to their excluder characteristics [20]. Still, some species may also be considered for phytoextraction in polluted agricultural soils presenting low to moderate Cd concentrations [7]. The use of grasses for phytoextraction or phytostabilization depends on many factors, such as their ability to translocate and tolerate Cd-induced stress. To screen high-biomass-producing grasses for their best use in enhancing phytoremediation efficiency and supporting the circular economy, certain indexes can be employed, such as the TE bioconcentration factor (BCF) and the TE translocation factor (TF) [22]. The Cd BCF is calculated by dividing the shoot Cd concentration by the available soil Cd concentration. In contrast, the Cd TF is calculated by dividing the shoot Cd concentration by the root Cd concentration [23]. It has been suggested that only species with both factors >1 have the potential for phytoextraction, while species selected for Cd phytostabilization should ideally have both BCF and TF values < 1 [22]. Given this context, the objective of this study was to evaluate the tolerance of high-biomass grasses by investigating both biomass production and nutrient accumulation and to screen them for phytoextraction or phytostabilization purposes in Cd-polluted soil by determining their Cd accumulation and calculating the Cd BCF and Cd TF.

## 2. Results

### 2.1. Biomass Production by Grasses Used for Cd Phytoremediation

**Figure 1:**
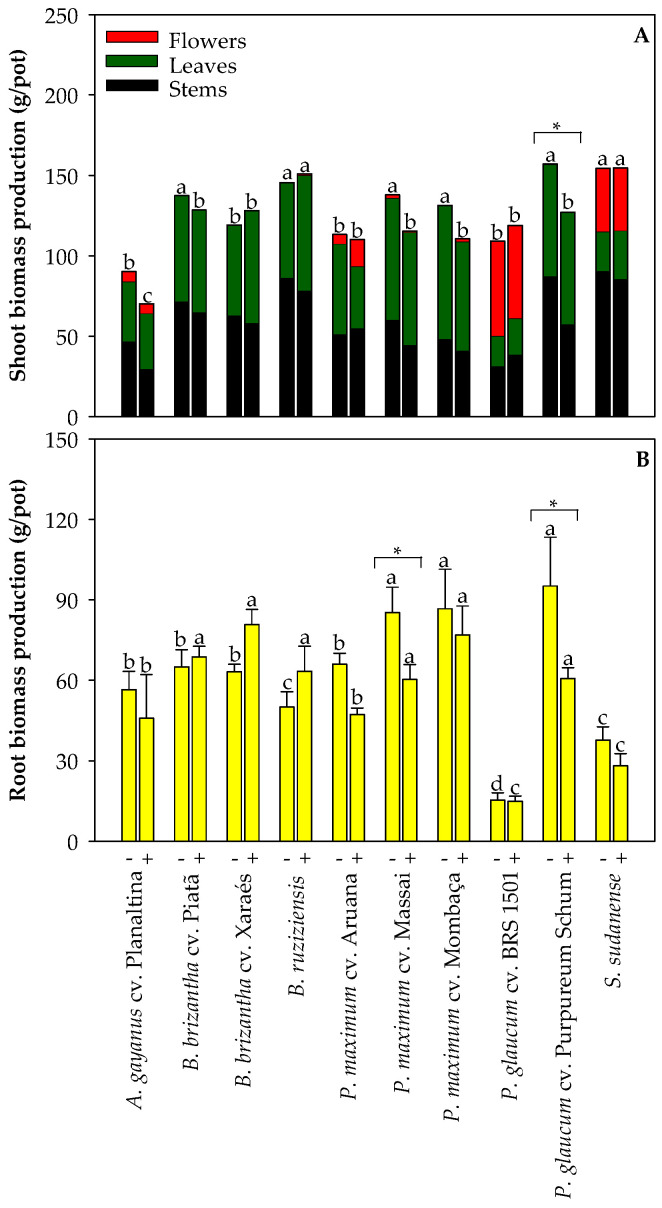
Shoot (**A**) and root (**B**) biomass production of grasses cultivated in non-polluted (-) and Cd-polluted (+) Oxisol. Shoot biomass includes stems, leaves, and flowers. Different letters on the bars indicate significant differences between grass species within each Cd pollution condition (Scott–Knott test, *p* < 0.05), and asterisks (*) indicate significant differences at *p* < 0.05 between Cd pollution conditions within each grass species (ANOVA, *t*-test).

**Figure 2:**
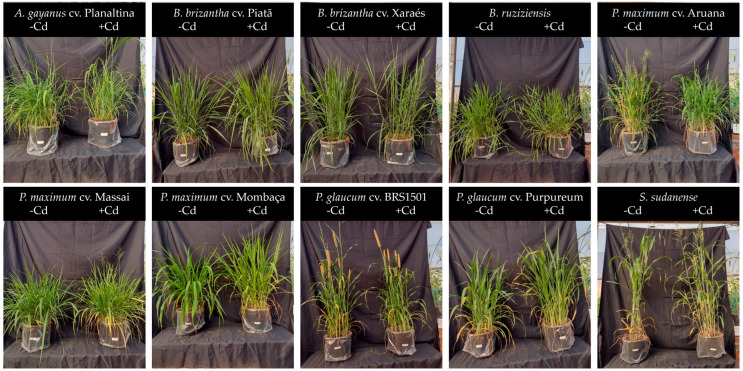
Pictures of the grass species cultivated in non-polluted (-Cd) and Cd-polluted (+Cd) Oxisol.

To evaluate the tolerance of high-biomass grass species to Cd exposure, we measured the biomass production of grasses grown in non-polluted and mildly Cd-polluted Oxisols (Figure 1 and Figure 2). *B. brizantha* cv. Piatã, *B. ruziziensis*, *P. maximum* cv. Massai, *P. maximum* cv. Mombaça, *P. glaucum* cv. Purpureum Schum, and *S. sudanense* produced the highest shoot biomass when grown in the non-polluted Oxisol (Figure 1A). When the grasses were grown in the Cd-polluted Oxisol, *B. ruziziensis* and *S. sudanense* presented the highest shoot biomass production; however, only *P. glaucum* cv. Purpureum Schum exhibited a reduction in the shoot biomass due to the exposure to Cd (Figure 1A). Regarding the root biomass production, *P. maximum* cv. Massai, *P. maximum* cv. Mombaça, and *P. glaucum* cv. Purpureum Schum presented the highest values when grown in the non-polluted Oxisol (Figure 1B). Conversely, when grown in the Cd-polluted Oxisol, *B. brizantha* cv. Piatã, *B. brizantha* cv. Xaraés, *B. ruziziensis*, *P. maximum* cv. Massai, *P. maximum* cv. Mombaça, and *P. glaucum* cv. Purpureum Schum exhibited the highest root biomass production. However, Cd exposure lowered root biomass only in *P. maximum* cv. Massai and *P. glaucum* cv. Purpureum Schum (Figure 1B). Cadmium exposure did not significantly affect the root/shoot ratio (data not shown).

### 2.2. Effect of Cd on Nutrient Concentration in the Grasses Used for Phytoremediation

**Figure 3:**
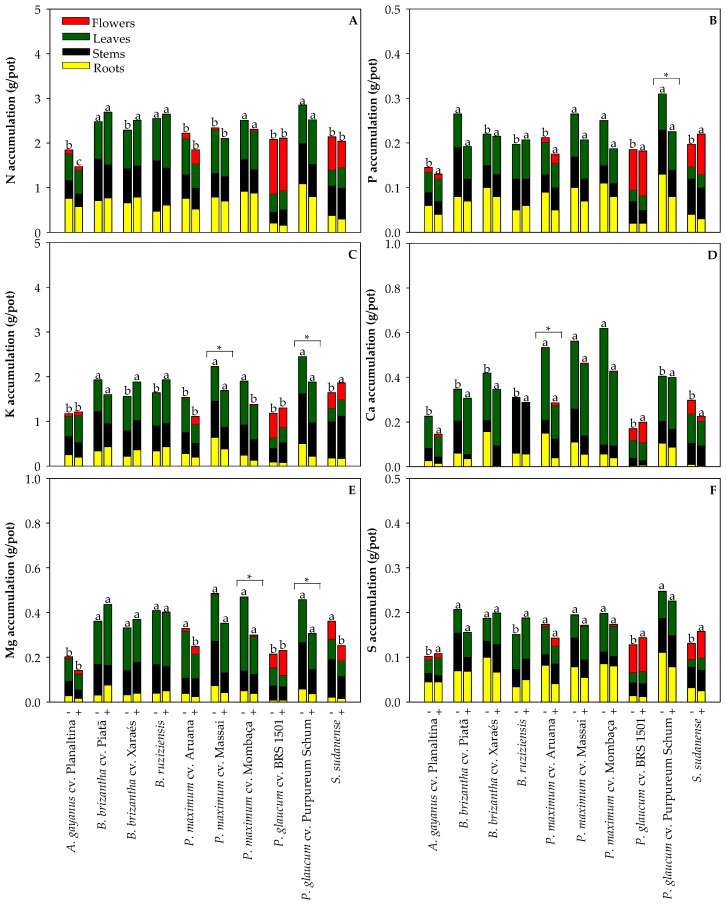
Accumulation of nitrogen—N (**A**), phosphorus—P (**B**), potassium—K (**C**), calcium—Ca (**D**), magnesium—Mg (**E**), and sulfur—S (**F**) in the roots, stems, leaves and flowers of grasses cultivated in non-polluted (-) and Cd-polluted (+) Oxisol. Different letters on the bars indicate significant differences between grass species within each Cd pollution condition (Scott–Knott test, *p* < 0.05), and asterisks (*) indicate significant differences at *p* < 0.05 between Cd pollution conditions within each grass species (ANOVA, *t*-test).

**Figure 4:**
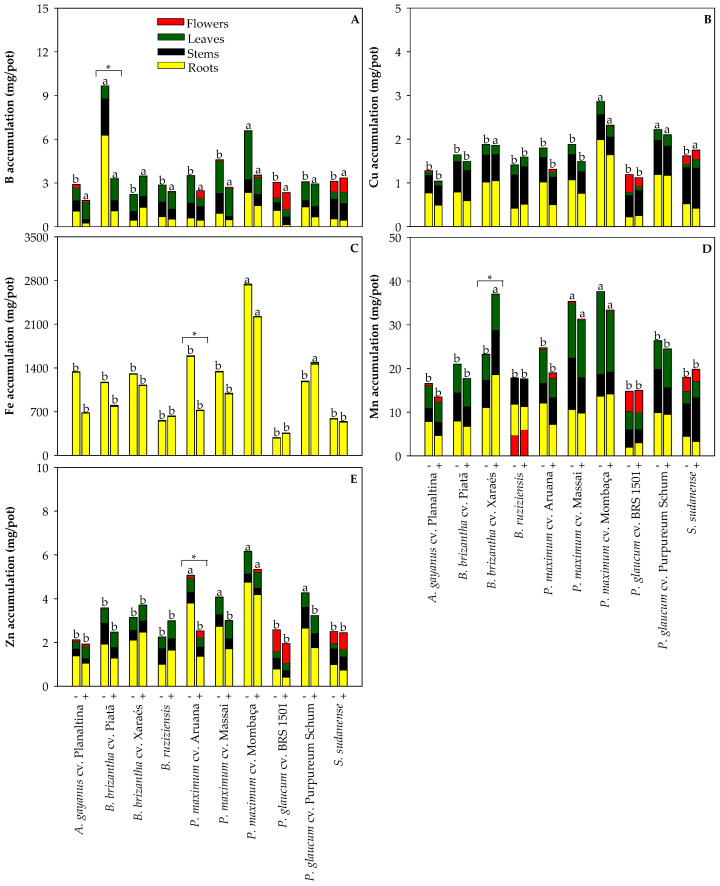
Accumulation of boron—B (**A**), copper—Cu (**B**), iron—Fe (**C**), manganese—Mn (**D**), and zinc—Zn (**E**) in the roots, stems, leaves, and flowers of grasses cultivated in non-polluted (-) and Cd-polluted (+) Oxisol. Different letters on the bars indicate significant differences between grass species within each Cd pollution condition (Scott–Knott test, *p* < 0.05), and asterisks (*) indicate significant differences at *p* < 0.05 between Cd pollution conditions within each grass species (ANOVA, *t*-test).

We investigated nutrient accumulation to determine if Cd exposure induced nutritional disorders in the assessed species. Differences were found for N, P, K, Ca, Mg, S (Figure 3), B, Cu, Fe, Mn, and Zn (Figure 4) accumulation when cultivated in a non-polluted and a Cd-polluted Oxisol. When cultivated in the Cd-polluted Oxisol, *P. glaucum* cv. Purpureum Schum demonstrated reductions in P, K, and Mg accumulation (Figure 3B,C,E), *P. maximum* cv. Massai showed lower K accumulation (Figure 3C), *P. maximum* cv. Mombaça exhibited lower Mg accumulation (Figure 3E), *P. maximum* cv. Aruana demonstrated reductions in Ca, Fe, and Zn accumulation (Figure 3D and Figure 4C,E), and boron decreased in *B. brizantha* cv. Piatã (Figure 4A). The only increase in nutrient accumulation due to Cd exposure was observed for Mn in *B. brizantha* cv. Xaraés (Figure 4D). Additionally, it is noteworthy that Fe accumulation in the roots of all tested grasses was very high, regardless of Cd exposure. This can be attributed to the formation of Fe plaques on the root surface due to the high Fe concentrations in Oxisols [24].

### 2.3. Cadmium Concentrations in the Oxisol and in the Tissues of Grasses Used for Phytoremediation

**Figure 5:**
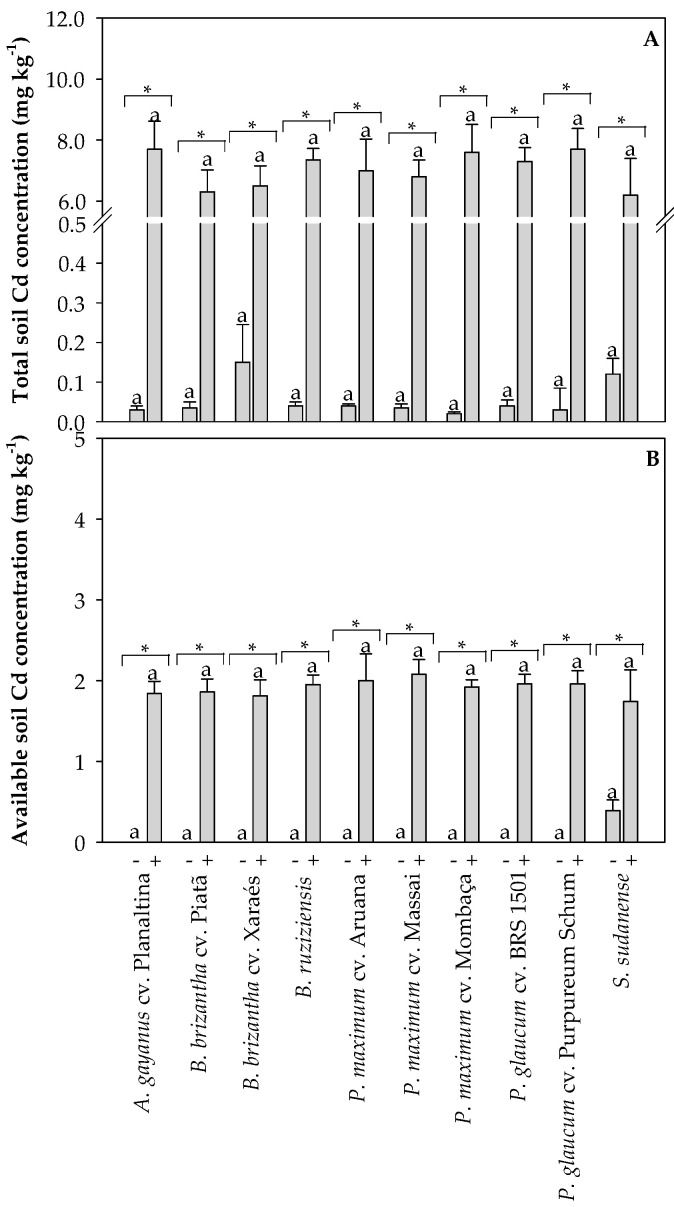
Total (**A**) and Mehlich-1 extractable soil Cd concentrations (**B**) measured in non-polluted (-) and Cd-polluted (+) Oxisol at the end of the experiment. Different letters on the bars indicate significant differences between grass species within each Cd pollution condition (Scott–Knott test, *p* < 0.05), and asterisks (*) indicate significant differences at *p* < 0.05 between Cd pollution conditions within each grass species (ANOVA, *t*-test).

**Figure 6:**
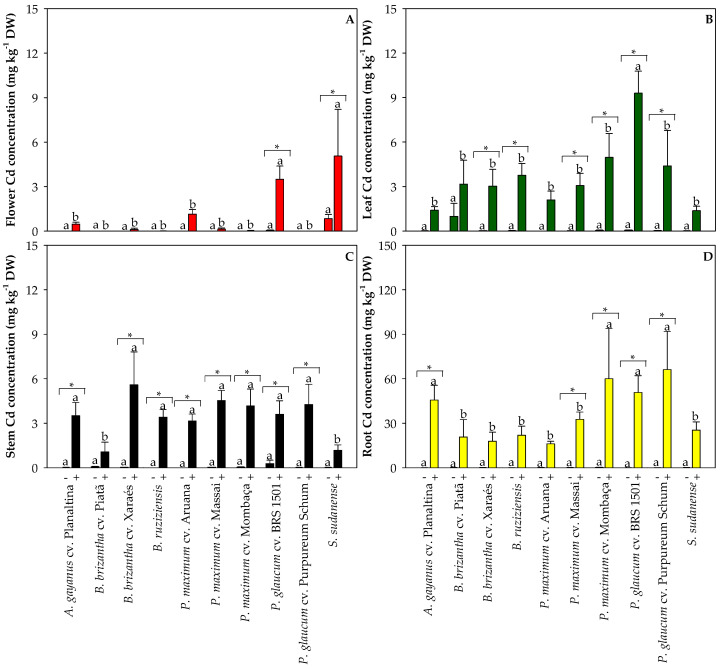
Cadmium concentrations in the flowers (**A**), leaves (**B**), stems (**C**), and roots (**D**) of grasses cultivated in non-polluted (-) and Cd-polluted (+) Oxisol. Different letters on the bars indicate significant differences between grass species within each Cd pollution condition (Scott–Knott test, *p* < 0.05), and asterisks (*) indicate significant differences at *p* < 0.05 between Cd pollution conditions within each grass species (ANOVA, *t*-test).

The total Cd concentration in the non-polluted Oxisol was below 0.2 mg kg^−1^, and about 7.5 mg kg^−1^ in the Cd-spiked Oxisol (Figure 5A). The ‘available’ Cd concentration in the Cd-spiked Oxisol was about 2 mg kg^−1^ (Figure 5B). Grasses cultivated in the non-polluted Oxisol did not differ in Cd concentration in the roots, stems, leaves, and flowers, with values below 1 mg kg^−1^ DW in all tissues (Figure 6). On the other hand, *P. glaucum* cv. BRS 1501 and *S. sudanense* had higher Cd concentrations in the flowers compared with the other species when grown in the Cd-polluted Oxisol (Figure 6A). *P. glaucum* cv. BRS 1501 exhibited the highest Cd concentration in the leaves when cultivated in the Cd-polluted Oxisol (Figure 6B). The highest Cd concentrations in stems were found in *A. gayanus* cv. Planaltina, *B. brizantha* cv. Xaraés, *B. ruziziensis*, *P. maximum* cv. Aruana, *P. maximum* cv. Massai, *P. maximum* cv. Mombaça, *P. glaucum* cv. BRS 1501, and *P. glaucum* cv. Purpureum Schum (Figure 6C). The highest Cd concentrations in roots were found for *A. gayanus* cv. Planaltina, *P. maximum* cv. Mombaça, *P. glaucum* cv. BRS 1501, and *P. glaucum* cv. Purpureum Schum (Figure 6D). A decreasing gradient of Cd concentrations was observed from roots to shoots, indicating a clear limitation of upward Cd transport. In general, the tissue Cd concentrations tended to be higher in grasses cultivated in the Cd-polluted Oxisol compared with non-polluted Oxisol (Figure 6).

### 2.4. Cadmium Accumulation and Factors Related to Phytoremediation Efficiency in Grasses

**Figure 7:**
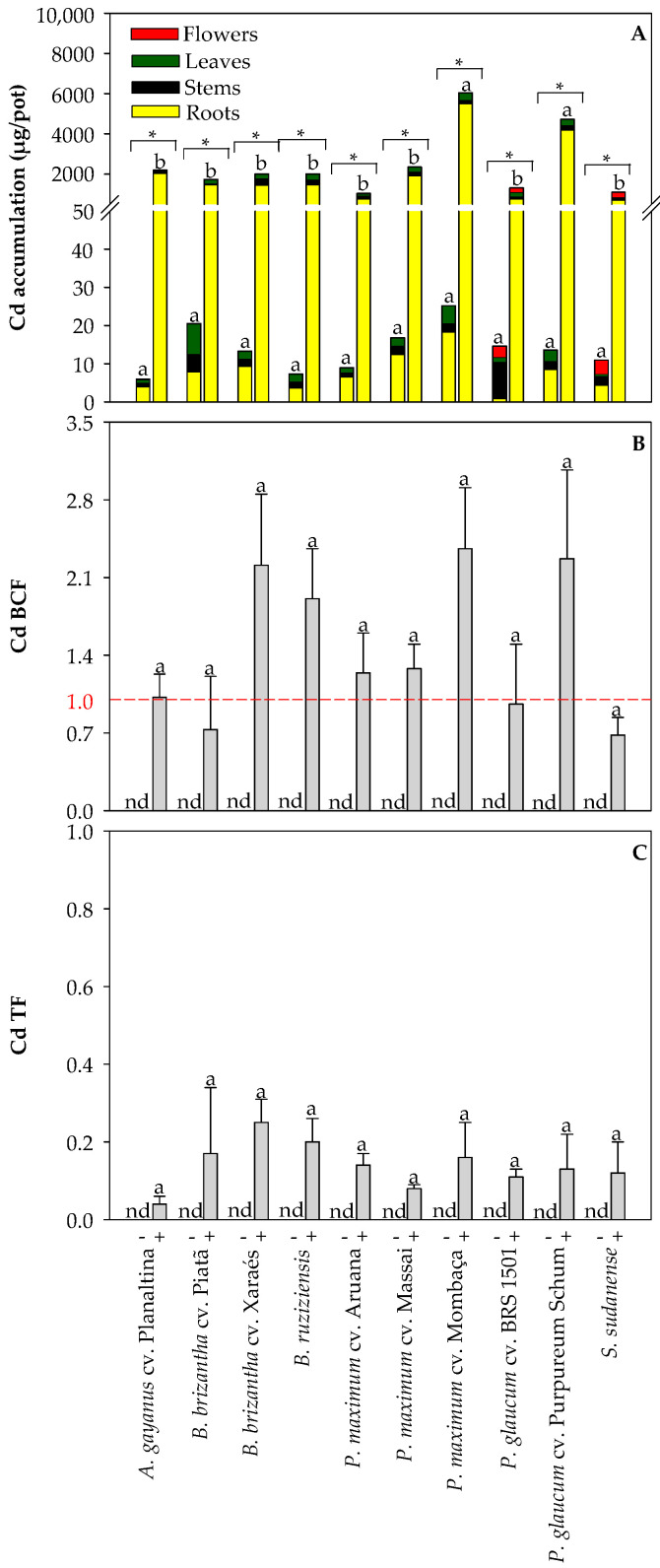
Cadmium accumulation in the root, stems, leaves, and flowers (**A**), Cd bioconcentration factor—Cd BCF (**B**) and Cd translocation factor—Cd TF (**C**) of grasses cultivated in non-polluted (-) and Cd-polluted (+) Oxisol. Different letters on the bars indicate significant differences between grass species within each Cd pollution condition (Scott–Knott test, *p* < 0.05), and asterisks (*) indicate significant differences at *p* < 0.05 between Cd pollution conditions within each grass species (ANOVA, *t*-test). nd: non-determined.

To screen the grasses for their suitability for phytoextraction or phytostabilization of Cd, we also determined Cd accumulation, Cd BCF, and Cd TF (Figure 7). The absorbed Cd accumulated predominantly in the roots rather than in the above-ground plant parts (Figure 7A). There were no significant differences in Cd accumulation between grasses grown in the non-polluted soil. Compared with the non-polluted soil, Cd accumulation was significantly higher in all grasses grown in the Cd-polluted soil (Figure 7A). *P. maximum* cv. Mombaça and *P. glaucum* cv. Purpureum Schum presented the highest Cd accumulation. Since calculation for non-polluted conditions does not make sense, the Cd BCF and Cd TF were calculated only for plants grown in the Cd-polluted soil. The grass species considered did not differ in their Cd BCF, but *B. brizantha* cv. Xaraés, *B. ruziziensis*, *P. maximum* cv. Aruana, *P. maximum* cv. Massai, *P. maximum* cv. Mombaça, and *P. glaucum* cv. Purpureum Schum exhibited a Cd BCF > 1 (Figure 7B). The different grass species also did not differ in Cd TF and all showed values lower than 0.4 (Figure 7C), indicating that Cd transport from roots to shoots was restricted.

## 3. Discussion

### 3.1. Tolerance of High-Biomass Grasses in a Cd-Polluted Oxisol

As the uptake and accumulation of Cd vary from plant to plant, the evaluation of new genotypes remains a focal point in current research endeavors to identify genotypes that are more tolerant to Cd-induced stress and, thus, their potential use in Cd-polluted soils [2]. An excess of Cd usually negatively affects plant growth and biomass production by disrupting cellular homeostasis, metabolic pathways, and ATP synthesis, as well as by inducing chromosomal aberrations and damages to mitochondria, inhibiting the formation of photosynthetic pigments and reducing photosynthetic efficiency, among other changes [25]. Therefore, from a simplistic point of view, it is possible to estimate the tolerance of plants to Cd by simply determining biomass production. We observed that the grass species under investigation differed in biomass production, which was expected due to their existing genomic variation. However, only *P. maximum* cv. Massai and *P. glaucum* cv. Purpureum Schum exhibited lower root biomass production upon Cd exposure (Figure 1). It means that most grasses assessed could deal with the soil Cd concentrations of 7.5 mg kg^−1^ by employing tolerance mechanisms, which could include but are not restricted to (i) limitation of Cd movement to roots by mycorrhizas; (ii) binding Cd to the cell walls and root exudates; (iii) reduced Cd influx across the plasma membrane; (iv) active Cd efflux into the apoplast; (v) Cd chelation in the cytosol followed by transportation and accumulation into the vacuoles; and (vi) maintaining the balance between the antioxidants and oxidants [7]. Although these processes are essential for grasses coping with Cd-induced stress, they restrict upward transport of Cd (Figure 6 and Figure 7), resulting in high Cd levels in the roots and basal nodes, which can lead to nutritional imbalances in the lower parts of plants used for Cd phytoremediation [21].

Cadmium exposure caused reductions in P, K, and Mg accumulation in *P. glaucum* cv. Purpureum Schum; K accumulation in *P. maximum* cv. Massai; Mg accumulation in *P. maximum* cv. Mombaça; Ca, Fe, and Zn accumulation in *P. maximum* cv. Aruana; and B accumulation in *B. brizantha* cv. Piatã (Figure 3 and Figure 4). The lower K accumulation in both *P. glaucum* cv. Purpureum Schum and *P. maximum* cv. Massai (Figure 3C) likely contributed to the lower root biomass production under Cd exposure (Figure 1B), as K is involved in the redistribution of photoassimilates and biomass allocation [26]. A recent study with *S. bicolor* pointed out that additional K fertilization was required to prevent Cd-induced reductions in biomass production [27]. Alterations in the nutrient concentrations are usually coupled with adverse effects on the development of plants under Cd stress, but adjustments in the ionomic profile are related to protective strategies to overcome the side effects of Cd accumulation [28]. If we consider that most changes in nutrient accumulation were not coupled with reductions in biomass production, it makes sense. On the other hand, over time, the lower nutrient accumulation in Cd-exposed grasses may impair plant development due to nutritional limitations [29]. Therefore, proper fertilization is essential to enhance the performance of Cd-exposed grasses cultivated [9,27,30].

### 3.2. Screening Grasses for Phytoextraction or Phytostabilization of a Cd-Polluted Oxisol

It is possible to group the species according to their Cd accumulation capability: (i) ‘standard’ plants (can only tolerate low concentrations of available Cd in the soil before they suffer due to acute phytotoxicity); (ii) excluders (can grow over a wide range of available Cd before physiological mechanisms cannot control and allow unregulated uptake, resulting in growth inhibition of the plant); (iii) bioindicators (take up Cd over a broader range than ‘standard’ plants and the concentrations in plant leaves reflect that of the soil until phytotoxicity inhibits the growth and eventually causes the death of the plant); and (iv) hyperaccumulators (able to withstand much higher concentrations of ‘bioavailable’ Cd than ‘standard’ plants, bioindicators and excluders) [31]. Grasses generally are classified as excluders or bioindicators [7,17,20], which means that these plants restrict the transport of metals from roots to shoots (as observed in this study; Figure 6 and Figure 7) and are more suitable for Cd phytostabilization. On the other hand, the greater biomass of these plants could enhance Cd removal from polluted soils due to higher Cd accumulation, which is desirable for phytoextraction purposes. Therefore, adequate screening and selection of plant species for Cd phytoremediation play an essential role in the development of remediation methods (decontamination or stabilization), especially in mildly to moderately polluted soils [32].

Some indexes, such as BCF and TF, have been employed to screen plants suitable for phytoextraction or phytostabilization [22]. Grasses with both BCF and TF > 1 have potential to be used for phytoextraction due to more significant Cd accumulation in the above-ground parts of plants. In contrast, species selected for Cd phytostabilization should ideally have BCF and TF values <1 [22]. In our study, the grass species assessed preferentially accumulated Cd in the roots (Figure 7A), presented a Cd BCF between 0.7 and 2.3 (Figure 7B), and presented a Cd TF below 0.4 (Figure 7C). Plants presenting BCF > 1 and TF < 1 have also been recommended for phytostabilization when they present tolerance to pollutant-induced stress [22]. A vigorous root system is important for plants used for Cd phytostabilization since Cd can be immobilized in soils through sorption by roots or accumulated into the roots [33]. As both *P. maximum* cv. Massai, and *P. glaucum* cv. Purpureum Schum presented lower root biomass production under Cd exposure (Figure 1B), the use of these grasses for phytostabilization is probably not feasible over time in soils presenting moderate soil Cd pollution. On the other hand, all other grass species assessed are good candidates for Cd phytostabilization in conditions of mild pollution. Also, in the case of Cd phytostabilization, the above-ground biomass produced by grasses is unsuitable for feeding animals. However, the biomass could be used for bioenergy production and high-value by-product generation, contributing to a circular economy [5].

## 4. Materials and Methods

### 4.1. Soil Collection and Characterization

The soil used in this study was collected from the upper layer (0.0–0.2 m depth), in an area with little anthropic influence, located in Lavras, Brazil (21°13′55.5″ S; 44°57′56.8″ W). The soil used is classified as Oxisol, according to Soil Taxonomy [34]. Soil characteristics were determined in air-dried soil sieved through a 2 mm mesh: pH (H_2_O) = 5.5, K (Mehlich-1) = 10.41 mg dm^−3^, P (Mehlich-1) = 0.10 mg dm^−3^, Na (Mehlich-1) = 2.00 mg dm^−3^, Ca (KCl 1 mol L^−1^) = 0.41 cmol_c_ dm^−3^, Mg (KCl 1 mol L^−1^) = 0.09 cmol_c_ dm^−3^, Al (KCl 1 mol L^−1^) = 0.10 cmol_c_ dm^−3^, H + Al (SMP extractor) = 1.50 cmol_c_ dm^−3^, Zn (Mehlich-1) = 0.10 mg dm^−3^, Fe (Mehlich-1) = 42.80 mg dm^−3^, Mn (Mehlich-1) = 9.90 mg dm^−3^, Cu (Mehlich-1) = 0.74 mg dm^−3^, B (hot water) = 0.06 mg dm^−3^, S (monocalcium phosphate in acetic acid) = 6.70 mg dm^−3^, organic matter (oxidation with Na_2_Cr_2_O_7_ 4N + H_2_SO_4_ 10N) = 0.55 dag kg^−1^, sum of bases (SB) = 0.53 cmol_c_ dm^−3^, effective cationic exchange capacity (CECe) = 0.63 cmol_c_ dm^−3^, total CEC (CECt) = 2.03 cmol_c_ dm^−3^, base saturation (V%) = 25.95%, and Al saturation (m%) = 15.87%. Granulometric fractions were obtained by the hydrometer method [35]: sand = 21 dag kg^−1^, silt = 12 dag kg^−1^, and clay = 67 dag kg^−1^.

### 4.2. Plant Material and Experimental Design

The species *Andropogon gayanus* cv. Planaltina, *Brachiaria brizantha* (Syn. *Urochloa brizantha*) cv. Piatã, *Brachiaria brizantha* (Syn. *Urochloa brizantha*) cv. Xaraés, *Brachiaria ruzi-ziensis* (Syn. *Urochloa ruziziensis*), Panicum maximum (Syn. *Megathyrsus maximus*) cv. Aruana, Panicum maximum (Syn. *Megathyrsus maximus*) cv. Massai, Panicum maximum (Syn. *Megathyrsus maximus*) cv. Mombaça, *Pennisetum glaucum* cv. BRS 1501, *Pennisetum glaucum* cv. Purpureum Schum, and *Sorghum sudanense* were grown under greenhouse conditions with natural light intensity, average photoperiod of 16 h, an average temperature of 25 °C and average relative humidity of 74%. Two Oxisol soil conditions were tested: non-polluted (control, with a total Cd concentration of 0.17 mg kg^−1^) and Cd-polluted. Soil Cd pollution was achieved by spiking Cd(NO_3_)_2_·4H_2_O to raise the total Cd concentration to 7.5 mg kg^−1^. This concentration was chosen considering the satisfactory growth of some grasses in soils presenting Cd concentrations close to this value [9,21]. Pots were arranged in a completely randomized design with four replicates per condition.

### 4.3. Soil Pollution, Growth Conditions, Plant Harvesting and Sampling

After collection, air-drying, characterization, and Cd spiking, a basal fertilization was performed following the recommendations for grasses [36] by applying 3.33 g of N (urea), 16.35 g of P (triple superphosphate), and 1.5 g of K (potassium chloride) per pot. Subsequently, the soils were incubated for 15 days at a soil moisture content of 70% of the maximum water-holding capacity [17]. After soil incubation, the seeds were sown in the pots. Following germination, periodic thinning was carried out until ten seedlings per pot were left. Thirty days after seed germination, 3.33 g of N (source) and 1.5 g of K (source) per pot were applied as topdressing [36]. Soil moisture content was kept constant at 70% of the maximum water-holding capacity throughout the study by adding deionized water. After ninety days of growth, plants were harvested and separated into roots, stems, leaves, and flowers + panicles (referred to as flowers in the text below) to determine biomass production and the concentrations of minerals and Cd. Soil samples were collected at the end of the study to assess the soil Cd concentrations.

### 4.4. Concentrations of Cd in the Soil

The total Cd concentration was determined by weighing 0.25 g of air-dried fine earth, which was placed in Teflon tubes, followed by the addition of 5 mL of concentrated acid (1:3 HCl/HNO_3_, *v*/*v*). The tubes were then placed in a microwave oven with controlled temperature and pressure, following the USEPA 3051a method [37]. After digestion, the samples were filtered through quantitative filter paper and diluted with ultrapure water to a final volume of 50 mL. The Cd concentration was measured using inductively coupled plasma optical emission spectrometry (ICP-OES, SPECTRO Analytical Instruments GmbH, model Blue, Kleve, Germany). Certified soil samples (BCR—142R—Light sandy soil) were used to ensure the quality of the analytical method.

The available soil Cd concentration was determined with the assistance of a beaker bong, in which 10 cm^3^ of air-dried fine earth was poured into a 125 mL Erlenmeyer flask, to which 100 mL of a Mehlich-1 extractant solution was added, consisting of 0.0125 mol L^−1^ of H_2_SO_4_ + 0.05 mol L^−1^ of HCl. After that, the solution was shaken for 5 min in a horizontal shaker (220 rpm) and the extract was left to rest for 16 h for decantation and collection of supernatants [38]. Subsequently, the extracts were analyzed by ICP-OES.

### 4.5. Minerals and Cd in the Plant Tissues

Plant tissues were dried in a forced ventilation oven at 65 °C for 72 h and then ground in a Wiley-type mill to determine the concentrations of N, P, K, Ca, Mg, S, B, Cu, Fe, Mn, Zn, and Cd. Total N was determined after sulfuric acid digestion by the Kjeldahl method using steam distillation [39]. For the determination of the other elements, the plant material was digested in a microwave oven using a mixture of HNO_3_ and H_2_O_2_, following the USEPA 3051a method [37]. The extracts were analyzed by ICP-OES. Blank reagent samples were used during digestion for quality control. Standard reference material (1570a—spinach leaves) was also used to ensure the accuracy and precision of the analytical methods. Element accumulation was calculated by multiplying the tissue’s element concentration by the respective tissue’s dry weight.

### 4.6. Calculation of Indexes Related to Phytoremediation

To screen the grasses in terms of their best use for phytoextraction or phytostabilization, Cd BCF was calculated by dividing the shoot Cd concentrations (average Cd concentrations in stems, leaves, and flowers) by the available soil Cd concentrations, and Cd TF was calculated by dividing the shoot Cd concentrations by the root Cd concentrations [23].

### 4.7. Statistical Analysis

Normality and homoscedasticity were checked before interpreting the results of the analysis of variance (ANOVA). Subsequently, with the validation of the ANOVA assumptions, the Scott–Knott test (*p* < 0.05) was used to compare means between grass species within each Cd pollution condition. Two-by-two comparisons were conducted using the *t*-test to compare the means between Cd pollution conditions within each grass species. Statistical analyses were performed using SISVAR^®^ v. 5.3 [40], and the graphs were created and plotted with SigmaPlot (v. 10.0, Systat Software Inc., San Jose, CA, USA). The results were expressed as means ± standard error of the mean (SEM).

## 5. Conclusions

Our findings indicate that biomass production in most grasses was not impaired by moderate Cd pollution, except for *P. maximum* cv. Massai and *P. glaucum* cv. Purpureum Schum, which showed reduced root biomass production. Such results suggest that *A. gayanus* cv. Planaltina, *B. brizantha* cv. Piatã, *B. brizantha* cv. Xaraés, *B. ruziziensis*, *P. maximum* cv. Aruana, *P. maximum* cv. Mombaça, *P. glaucum* cv. BRS 1501, and *S. sudanense* were able to cope with the stress caused by soil Cd concentrations of 7.5 mg kg^−1^. Cadmium exposure altered nutrient accumulation in some grass species. However, this was not associated with lower biomass production, except for K accumulation, which was associated with reduced root biomass allocation in *P. maximum* cv. Massai and *P. glaucum* cv. Purpureum Schum. The changes observed in nutrient accumulation likely occurred as a strategy to adjust the Cd tolerance mechanisms of the grasses; however, this assumption requires further investigation. All grass species showed restricted upward Cd transport and high Cd accumulation in the roots. Although some grasses exhibited a Cd BCF > 1, the Cd TF for all species was below 0.4. Altogether, the results indicate that the grass species assessed are more suitable for Cd phytostabilization than Cd phytoextraction under conditions of moderate Cd pollution, except for *P. maximum* cv. Massai and *P. glaucum* cv. Purpureum Schum, which showed reduced root biomass production and may not be efficient over time.

## Data Availability

Data are contained within the article. Additional information will be made available by the authors on request.
